# Supplementary material for: Characterization of the Diagnostic Performance of a Novel COVID-19 PETIA in Comparison to Four Routine N-, S- and RBD-Antigen Based Immunoassays
Source: Diagnostics (Basel). 2021 Jul 25;11(8):1332. doi: 10.3390/diagnostics11081332 (PMC8394885; doi:10.3390/diagnostics11081332)

Supplementary Table S1: Overview of the specification of SARS-CoV-2 immuno assays. Information depicted as claimed by respective manufacturers within their assay manuals and package inserts. TtR (time to result).

**Manufacturer:**	**DiaSorin**	**DiaSys**	**EUROIMMUN**	**ROCHE**	**ROCHE**
**Assay name:**	LIAISON® SARS-CoV-2 S1/S2 IgG	SARS-CoV-2 UTAB FS	Anti-SARS-CoV-2 ELISA (IgG)	Elecsys® Anti-SARS-CoV-2	Elecsys® Anti-SARS-CoV-2 S
**Method:**	CLIA	PETIA	ELISA	ECLIA	ECLIA
**Immunoglobulin:**	IgG	IgG / IgM / IgA	IgG	IgG / IgM / IgA	IgG / IgM / IgA
**Antigen:**	S1 and S2 domain of spike protein (S1/S2)	Spike receptor binding domain (RBD)	S1 domain of spike protein, including RBD	Nucleocapsid protein (N)	Spike receptor binding domain (RBD)
**Version/Lot used:**	354021	E056-057/20	E201009AQ	51233900	51062300
**Quantification:**	quantitative	quantitative	semiquantitative	qualitative	quantitative
**Calibrator:**	2-level, liquid (Lot: 354021)	4-level, liquid (Lot: 293648)	1-level, liquid	2-level, liquid (Lot: 51233901)	2-level, liquid (Lot: 51047101)
**Controls:**	2-level, liquid (Lot: 357014)	2-level, liquid (Lot: E482/20; Lot: 293638)	2-level, liquid	2-level, liquid	2-level, liquid (Lot: 51046701)
**sample (µL):**	20	13	10	20	20
**TtR (min):**	35	11	150	18	18

Supplementary Table S2: Patient serum samples with various antibody titers and their respective *SARS-CoV-2 UTAB FS* results.

**Routine samples**	***SARS-CoV-2 UTAB FS* [AU/ml]**	**positive serology**										
		**CHPN IgM**	**CHPN IgG**	**MUM IgG**	**VZV IgA**	**VZV IgG**	**MAS IgG**	**CMV IgM**	**CMV IgG**	**VCA IgM**	**VCA IgG**	**EBNA IgG**
1	0.00								+			
2	0.00								+		+	+
3	26.06								+		+	+
4	10.88								+		+	+
5	0.00	+	+									
6	0.00		+									
7	0.00		+									
8	0.00										+	+
9	0.00											
10	0.00				+	+		+	+		+	
11	0.00			+		+	+					
12	0.00			+		+	+					
13	0.00			+		+	+					
14	0.00						+					
15	0.00					+	+					
16	0.00								+			
17	0.00					+			+		+	+
18	0.00											

Supplementary Table S3: Serum samples from children and pregnant women collected before the SARS-CoV-2 outbreak and their respective *SARS-CoV-2 UTAB FS* results.

**Pregnant women samples**	***SARS-CoV-2 UTAB FS* [AU/ml]**	**positive serology**										
		**CHPN IgM**	**CHPN IgG**	**MUM IgG**	**VZV IgA**	**VZV IgG**	**MAS IgG**	**CMV IgM**	**CMV IgG**	**VCA IgM**	**VCA IgG**	**EBNA IgG**
1	0.00		+	+			+		+		+	+
2	43.73	+	+			+	+		+		+	+
3	10.96		+	+		+	+		+	+	+	+
4	2.07		+			+			+		+	+
5	0.00		+	+		+	+				+	+
6	77.53			+		+	+				+	+
7	0.00	+	+	+							+	+
8	21.18			+		+	+		+		+	+
9	8.51		+	+		+	+		+		+	+
10	0.00			+		+	+		+		+	+

**Children samples**	***SARS-CoV-2 UTAB FS* [AU/ml]**
1	0.00
2	0.00
3	0.00
4	0.00
5	0.00
6	0.00
7	0.00
8	0.00
9	5.02
10	4.21

Supplementary Figure S1: Scatter diagrams of the samples (n = 222) measured with the five tests involved in the study. Roche-RBD (S) was arbitrarily used as reference. Values "lower than X (<X)" were given as X. The same was done for the values "higher than Y (>Y)", given as Y. The Scatter diagram function of MedCalc® Version 18.10.2 – 64-bit (MedCalc Software Ltd, Belgium) was employed for data representation. **A:** Euroimmune vs. Roche-RBD (S), Spearman´s R = 0.830; **B:** DiaSys vs. Roche-RBD (S), Spearman´s R = 0.863; **C:** Diasorin vs. Roche-RBD (S), Spearman´s R = 0.881; **D:** Roche-N vs. Roche-RBD (S), Spearman´s R = 0.872. However, this analysis has to be considered with caution for different reasons. Firstly, only three out the five evaluated methods were quantitative. Secondly, none of them was standardized at the time of measurements. Finally, no information is available about the production process and the parameters of the antigens employed by the manufacturers, what could potentially have a strong impact on their structural con-formation and immunoreactivity.

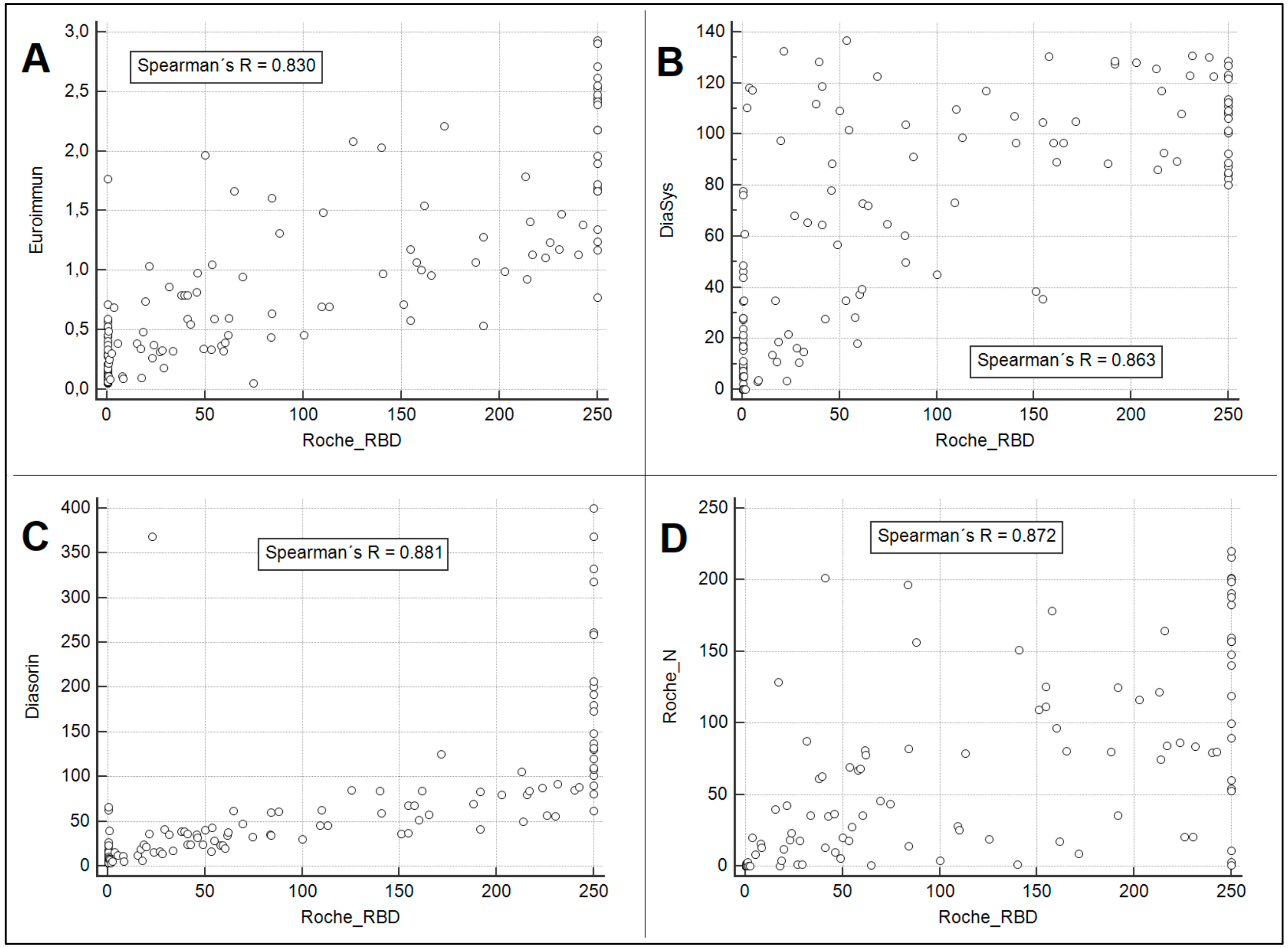

Supplement: Supplementary file 1 [file diagnostics-11-01332-s001.zip › diagnostics-1282314-supplementary.pdf]
